# Pediatric double-seropositive anti-glomerular basement membrane antibody disease: A case report and literature review 

**DOI:** 10.5414/CNCS111439

**Published:** 2024-11-14

**Authors:** Nadia Echcharii, Soukaina Essadiqi, Nabila Chekhlabi, Rajaa Tissir, Halima Hadri, Abire Allaoui, Nezha Dini

**Affiliations:** Department of Pediatrics, Cheikh Khalifa International University Hospital, Faculty of Medicine, Mohammed VI University of Health Sciences (UM6SS), Casablanca, Morocco

**Keywords:** pediatrics, anti-GBM disease, ANCA, anti-GBM, Goodpasture disease, kidney, rituximab

## Abstract

Introduction: Glomerular basement membrane (GBM) disease is a severe and exceedingly rare disorder characterized by the presence of circulating antibodies targeting the non-collagen NC1 domain of the α3 chain of collagen type IV in glomerular and alveolar basement membranes. It typically presents as rapidly progressive glomerulonephritis (RPGN), often accompanied by pulmonary hemorrhage. The occurrence of double-seropositivity for anti-GBM antibody and anti-neutrophil cytoplasmic antibody (ANCA), primarily with myeloperoxidase specificity (MPO-ANCA), is particularly uncommon in pediatric cases. Case presentation: A 9-year-old boy was admitted to the pediatric ward exhibiting macroscopic hematuria, proteinuria, and acute kidney injury, with a gradual decline in kidney function. Pulmonary function remained normal. Circulating anti-GBM antibodies and ANCA, specifically targeting myeloperoxidase (MPO), were detected. Diagnosis was confirmed via percutaneous renal biopsy, which revealed circular glomerular crescents in 9 out of 16 glomeruli. Immunofluorescence examination exhibited a linear staining pattern of the capillary wall for IgG. Treatment involved 5 boluses of methylprednisolone, followed by prolonged oral prednisone, 11 plasma exchange sessions, and initiation of rituximab due to a moderate response to therapy. Subsequently, the patient’s condition significantly improved, with normalized renal function observed 24 months post treatment. Conclusion: Despite limited literature on pediatric anti-GBM and double-positive disease, it is imperative to consider these diagnoses in pediatric patients presenting with RPGN. This article offers a comprehensive summary of the main characteristics of this disease in children and emphasizes therapeutic approaches through a review of identified cases in individuals under 18 years of age.

## Introduction 

Anti-glomerular basement membrane (GBM) disease is a severe and exceptionally rare disorder, classified as a vasculitis affecting small vessels with immune complexes [1]. Characterized by the presence of circulating antibodies directed against the non-collagen NC1 domain of the alpha3 chain of collagen type IV in glomerular and alveolar basement membranes [2], it affects the glomerular capillaries, pulmonary capillaries, or both, with GBM deposition of anti-GBM autoantibodies. Lung involvement results in pulmonary hemorrhage, while kidney involvement causes glomerulonephritis characterized by necrosis and crescents [3]. In the absence of prompt treatment, the disease swiftly progresses to chronic kidney failure, resulting in a permanent loss of kidney function and a fatal outcome in approximately half of the patients [4]. 

The estimated incidence of Anti-GBM disease in adults is less than 0.5 – 1 per million per year, and it is even rarer in children. Nonetheless, it accounts for 10-15% of all cases of Rapidly Progressive Glomerulonephritis (RPGN) and 3% of crescentic GN in children [5]. 

Double-seropositivity for anti-GBM antibody and anti-neutrophil cytoplasmic antibody (ANCA), most commonly with myeloperoxidase specificity (MPO-ANCA), is rare in the pediatric population [6, 7], with only 11 cases documented in the literature. 

In this article, we present the case of a 9-year-old boy with double-seropositive anti-GBM disease, accompanied by a comprehensive review of all identified cases of anti-GBM antibody disease in children under 18 years of age. 

## Materials and methods 

We conducted a comprehensive search using the following keywords: Glomerular Basement Membrane Disease, Anti-Glomerular Basement Membrane Disease, anti-GBM, Anti-Neutrophil Cytoplasmic Antibody Associated Vasculitis, Goodpasture disease, Goodpasture syndrome, anti-glomerular basement membrane antibody disease, and pneumo-renal syndrome in children. The search spanned from 1981, marking the inception of reported cases, to 2022. 

We utilized databases such as MEDLINE, PubMed, Google Scholar, and MalaCards. Only articles written in English were included in our review. We focused on observations of patients under 18 years of age with confirmed anti-GBM antibody disease through kidney biopsy and/or serology. Cases with unspecified diagnostic methods or incomplete records were excluded from the analysis. A summary of pertinent information regarding the cases is presented in (Table 1). 

## Case presentation 

A 9-year-old boy presented to the Department of Pediatrics with a 10-day history of macroscopic hematuria, which had worsened in the last 24 hours. He also reported weight loss and anorexia. Notably, his mother had a history of multiple sclerosis. 

Upon admission, the boy was apyretic, with a weight of 28 kg and a height of 147 cm. His respiratory rate was 22 breaths per minute, SpO_2_ was at 96%, pulse rate was 105 beats per minute, and blood pressure (BP) measured 100/60 mmHg. Bilateral lung auscultation revealed breath sounds. Urine sediment analysis indicated hematuria (4+), proteinuria (2+), and leukocyturia without bacteria. There were no signs of skin rash, joint involvement, petechiae, or cyanosis. 

Laboratory results showed anemia (Hg 10 g/dL), leukocytosis (12,000/μL) with 80% neutrophils, thrombocytosis (428,000/μL), elevated C-reactive protein (1,12 mg/dL), ferritin (365 Ng/mL), and acute kidney injury (creatinine 2.36 mg/dL, estimated glomerular filtration rate (eGFR) 25 mL/min/1.73m^2^). Urine analysis revealed hematuria (128,103/mm^3^), leukocyturia (9,103/mm^3^), and a proteinuria/creatinuria ratio of 1.55 g/g. Plasma C3 and C4 levels were within normal range, and antinuclear antibodies and anti-DNA antibodies were negative. A chest CT scan showed normal lung findings. 

**Figure 1:**
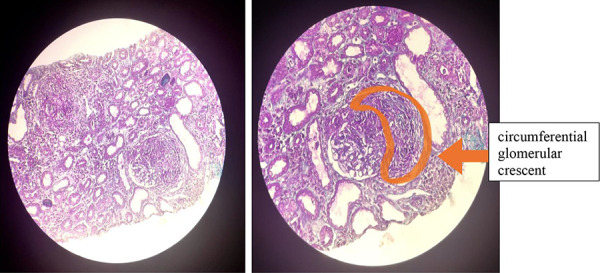
Light microscope sections of the patient’s renal biopsy revealing proliferative extra-capillary glomerulonephritis lesions with cellular crescents.

**Figure 2:**
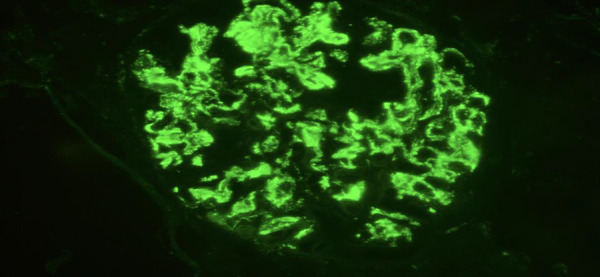
Immunofluorescence section of the patient’s renal biopsy revealing linear fixation of IgG along the glomerular basement membrane.

Despite close monitoring, kidney function deteriorated rapidly, with creatinine levels increasing to 3.79 mg/dL and eGFR dropping to 16 mL/min/1.73m^2^ by day 5. A prompt kidney biopsy revealed glomeruli with active circular crescents in 9 out of 16 glomeruli, accompanied by moderate chronic interstitial inflammation with no fibrosis. Minimal epithelial necrosis and rupture of Bowman’s capsule were observed in one glomerulus (Figure 1). Immunofluorescence examination demonstrated classical global and bright linear capillary wall staining for IgG without further deposition (Figure 2). The circulating anti-GBM autoantibody titer was markedly elevated at 680 IU/mL (normal < 6 IU/mL). Indirect immunofluorescence (IIF) revealed perinuclear anti-neutrophil cytoplasmic antibodies (p-ANCA) with specificity to myeloperoxidase (MPO) at a titer of 1 : 80. 

Immediate treatment ensued after the kidney biopsy, comprising 3 methylprednisolone pulses (1 g/1.73m^2^), prolonged oral prednisone (2 mg/kg/day), and 1 cyclophosphamide pulse (500 mg/m^2^ of body surface). Plasma exchange commenced after anti-GBM results and pulses, with a total of 11 sessions initially scheduled daily, then on alternating days. 

**Figure 3:**
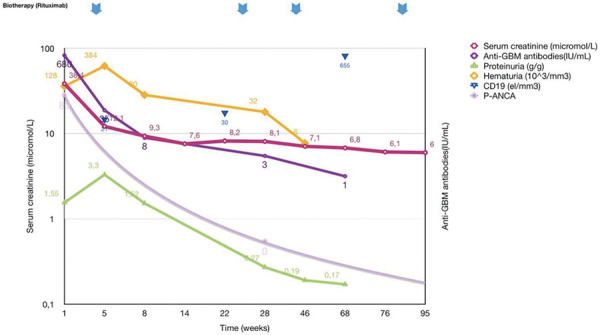
Treatment and follow-up of the patient up to 24 months.

Two weeks later, upon discontinuation of plasmapheresis, kidney function worsened with hyperkalemia, elevated blood urea level (1.76 g/L), oliguria, and generalized edema, accompanied by an increase in proteinuria to 2.2 g/g. Three hemodialysis sessions were administered, along with 2 additional methylprednisolone boluses and 2 units of whole blood transfusion due to anemia. Subsequently, rituximab (375 mg/m^2^) was initiated once a week for 4 weeks, resulting in a gradual improvement of symptoms. The estimated glomerular filtration rate (eGFR) increased from 16 to 52 and then 79 mL/min/1.73m^2^ at 6 months, with autoantibodies decreasing to under 7 IU/mL (Figure 3). 

At 6 months, persistent abnormal proteinuria led to the initiation of enalapril (3 mg/day, 0.2 mg/kg/day). Following a CD19 cell rebound after 16 months, a new session of rituximab was administered. At the last follow-up, 24 months after onset, the boy exhibited no clinical signs of disease activity, and anti-GBM levels remained undetectable. Additionally, MPO-ANCA was negative in the serum. Kidney function remained stable (eGFR 101 mL/min/1.73m^2^), and steroids were withdrawn 12 months after onset, without complications or significant side effects from treatment (Figure 3). Written consent was obtained from the parents. 

## Discussion and review of the literature 

Anti-GBM antibody disease is a rare autoimmune condition categorized as small vessel vasculitis in the Revised Chapel Hill International Consensus Conference of 2012 [1]. The term “Goodpasture disease” was coined in 1958 by Stanton and Tange to describe the pneumo-renal syndrome initially reported by Ernest Goodpasture in 1919 [8]. This term specifically denotes kidney damage, while “Goodpasture syndrome” is employed when referring to concurrent pneumo-renal damage. The term “anti-GBM antibody disease” encompasses both definitions and is the official nomenclature used in the literature [9]. 

In contrast to adults, pediatric cases of anti-GBM antibody disease are considerably scarce in the literature. There are 47 documented cases of anti-GBM antibody disease (Table 1) and only 12 cases of double-positivity. The distribution across all age groups is bimodal, with one peak occurring at a young age, predominantly in males, and a second peak in the 6^th^ – 8^th^ decades, where females predominate. Anti-GBM antibody disease is observed across all racial groups but is most prevalent in European Caucasians [10]. Among the 47 pediatric cases reported, there were 29 girls and 18 boys, resulting in a sex ratio of 0.62. The average age at diagnosis in children is 9.2 ± 4.6 years, with cases ranging from 11 months to 17 years. While the etiology of anti-GBM antibody disease remains unknown, it is hypothesized to involve a combination of environmental and genetic factors, triggering both humoral and cellular autoimmune responses [11]. 

Anti-GBM antibodies target the NC1 domain of the α3 chain of type IV collagen, primarily binding to the amino-terminal region of the NC1 domain. Recent studies have identified specific antibodies directed against the α5 NC1 domain, detected in 70% of anti-GBM antibody disease patients and linked to unfavorable kidney outcomes [12, 13]. Notably, despite the frequent reactivity of antibodies against other NC1 domains of collagen IV, only those against the α3 and α5 NC1 domains deposit in the basement membrane of the kidneys and lungs, suggesting their pivotal role in the pathogenesis of anti-GBM antibody disease [11, 12]. 

The predominant class of autoantibodies deposited along the GBM is IgG, with exceptionally rare reports of IgA or IgM, classifying them as atypical anti-GBM disease [14]. All four IgG subclasses have been detected in serum and kidney tissues, with IgG1 being the most common. Notably, IgG3 deposition correlates with the infiltration of inflammatory cells and kidney damage [14, 15]. 

A strong association exists between human leukocyte antigen (HLA) and anti-GBM disease. Genetic studies have demonstrated a robust connection with HLA-DR15, carrying a relative risk of ~ 8.5. In contrast, HLA-DR1, HLA-DR7, and HLA-DR9 are underrepresented, suggesting a potential protective role against the disease [16, 17, 18]. The pathogenesis of autoimmune diseases is believed to involve environmental factors in genetically predisposed individuals, leading to dysregulation between the innate and adaptive immune systems. Triggers such as smoking, hydrocarbon inhalation, and viral or bacterial infections have been implicated [19]. 

The coexistence of anti-GBM antibodies and antineutrophil cytoplasmic antibodies (ANCA) is rare in children. The genetic factors associated with this co-development are increasingly well-described, with associations reported for HLA-DRB1*1501, still classifying double positivity as a variant of anti-GBM disease [6]. Previous studies suggest that detectable ANCA may precede the development of anti-GBM antibodies, indicating a potential contribution of ANCA-mediated glomerular inflammation to the evolution of anti-GBM disease. Conversely, aberrant extracellular expression of myeloperoxidase has been linked to the development of anti-MPO antibodies, suggesting a reciprocal relationship [20]. 

Patients with double positivity (anti-GBM antibodies and ANCA) may have a more favorable kidney prognosis compared to isolated anti-GBM disease, showing an increased likelihood of kidney recovery and better long-term kidney survival [21, 22]. However, the presence of MPO-ANCA in double-positive patients is strongly associated with a higher risk of relapse. This association may be attributed to peroxidasine, an enzyme with a 48% similar structure to MPO-ANCA, and anti-peroxidasine antibodies are found at a significant rate in patients with double positivity [6, 23]. 

The clinical presentation is similar in double-positive patients and those with anti-GBM antibodies alone. The principal clinical features involve the development of kidney failure due to rapidly progressive glomerulonephritis (GN) or pulmonary hemorrhage [6, 14]. Among the 47 pediatric cases, 20 exhibited kidney involvement alone (42%), while two patients had isolated pulmonary involvement. In the pediatric series, general manifestations were observed, with pallor reported in 44% of cases, fever and lethargy in 41%, hematuria in 39%, nausea and vomiting in 30%, cough in 26%, lumbar and abdominal pain in 24%, edema in 24%, and high blood pressure in 15% (Table 1). 

Kidney disease typically progresses rapidly, with proteinuria and/or hematuria being the most common renal symptoms, occurring in 19 (40%) of the cases. The urine sediment may show microscopic or macroscopic hematuria, and 2 pediatric cases were discovered through a school urine screening program [24]. Progressive kidney failure may develop, leading to oliguria, fluid overload, and hypertension. 

Hemoptysis and coughing were the most common pulmonary symptoms, with cough reported in 12 (26%) of the cases and hemoptysis in 7 (15%). Pulmonary involvement was observed in 26 (55%) cases, with only two patients having isolated pulmonary involvement, although kidney biopsy was not performed in these cases. The incidence of alveolar hemorrhage is lower in double-positive patients, and isolated lung involvement appears to be more common in smokers [25]. 

Diagnosing anti-GBM disease requires the demonstration of anti-GBM antibodies in either the serum or the kidney. These antibodies are typically IgG1 and can be easily detected by enzyme-linked immunosorbent assay (ELISA) or Western Blot, both highly sensitive (> 95%) and specific (91%) [26, 27]. In the pediatric series, anti-GBM titers were positive in all patients tested (45/45), with only one patient showing an indeterminate result. Anti-MPO titer was positive in 12 out of 33 patients in whom it was assessed. Notably, our patient had both anti-MPO and anti-GBM positive titers. 

Kidney biopsy is crucial for diagnosis, even in the absence of anti-GBM antibodies when there is a strong suspicion of anti-GBM antibody disease [28, 29]. The biopsy provides valuable information on the activity and chronicity of kidney involvement, aiding in therapeutic decision-making [27]. Optical microscopy reveals extracapillary proliferation with cellular crescents, sometimes associated with foci of necrosis. Bowman’s capsule rupture is an indicator of severity [9, 14]. Immunofluorescence exhibits linear or non-linear deposits of immunoglobulin G along the glomerular basement membrane, which is pathognomonic for the disease, even in cases with normal kidney function. Deposits of C3, IgA, and IgM may be found in the distal tubules. The percentage of extraglomerular crescents appears correlated with IgG deposition and blood creatinine levels [9, 14]. Kidney biopsy was performed in 41 out of 47 pediatric cases, all concluding with glomerulonephritis and crescent formation. Our patient’s biopsy highlighted 9G/14, presenting an extracellular crescent (64%). It indicated the recent nature of kidney involvement with no fibrosis or tubular atrophy. However, the presence of a ruptured Bowman’s capsule and epithelial necrosis in certain tubules signified severity. Immunofluorescence confirmed the diagnosis of anti-GBM antibody disease, showing linear IgG deposits. 

The treatment of anti-GBM antibody disease necessitates urgent initiation even before confirming the diagnosis, as untreated cases progress rapidly to chronic kidney failure. In the absence of a pediatric consensus, the management in children is derived from the approach established for adults, as outlined in the KDIGO glomerular disease guidelines (2021) [30, 31]. Notably, there was no detectable difference between single-positive anti-GBM and double-positive groups regarding the initial treatment administered [6]. 

The therapeutic regimen is centered on purifying autoantibodies from the circulation through acute apheresis. Simultaneously, prevention of autoantibody production is achieved with cyclophosphamide and/or B-cell depleting agents. Glomerular inflammation is reduced with corticosteroids and/or adjunctive immune-modifying agents. Supportive treatment, including kidney replacement therapy, usually hemodialysis, is implemented if needed [31]. 

Immunosuppression consists of starting by pulse methylprednisolone (15 – 30 mg/kg up to a maximum dose of 1 g) daily for three doses, followed by daily oral prednisone (1 mg/kg per day to a maximum of 60 to 80 mg/day) for 1 month with gradual reduction over 6 to 12 months [31]. Cyclophosphamide is administered at a dose of 2 mg/kg/d orally for 3 months or intravenously as a bolus at a dose of 500 – 750 mg/m^2^ every 2 weeks for a total of 6 sessions or a monthly bolus for 2 – 3 months [32]. 

Out of the 47 pediatric patients identified, all underwent corticosteroid therapy, except for 1 case resulting in a fatal outcome on the 1^st^ day. Our patient, after not responding to the initial 3 boluses of methylprednisolone, received a total of 5 boluses. Additionally, 40 patients (85%) received cyclophosphamides, while our patient received a single bolus due to the favorable evolution before the 2^nd^ bolus and to avoid the side effects of cyclophosphamide. 

Plasmapheresis should be initiated as early as possible and continued until circulating anti-GBM antibodies turn negative. Apheresis initiation is recommended for GBM antibody disease, but caution is exercised in patients with serum creatinine greater than 5.7 mg/dL or dependent on dialysis, except in cases of intra-alveolar hemorrhage [33]. Combination plasmapheresis/immunosuppression has shown greater efficacy compared to immunosuppression alone [34, 35]. Apheresis techniques include plasma exchange, immunoadsorption, and double filtration plasmapheresis (DFPP). Dorval et al. [36] reported the first pediatric case treated with a combination of immunoadsorption (AI) and immunosuppressants with immediate and lasting improvement. In the pediatric series, 80% of patients received plasmapheresis. 

Rituximab, an anti-CD20 monoclonal chimeric antibody inducing peripheral B-cell depletion, is employed in severe cases to induce prolonged remission [37, 38]. 

KDIGO recommends using rituximab as an induction treatment instead of cyclophosphamides in children and pre-adolescents in case of ANCA vasculitis, and as maintenance treatment to sustain depletion in double-positive patients [31, 39, 40]. 

Efficacy of depletion lasts 6 – 8 months for most patients, sometimes up to 18 months [40]. 

Biotherapy with rituximab has demonstrated efficacy in managing relapses with fewer associated side effects [40]. In our patient’s scenario, rituximab was initiated following a worsening condition and the initial lack of response to corticosteroid therapy and cyclophosphamides. The primary objective was to preserve fertility. Among the 47 documented pediatric cases, only 7 individuals experienced positive outcomes with rituximab biotherapy, with our patient representing the 8^th^ case. 

Mycophenolate mofetil has been used successfully for disease induction and maintenance in a small number of patients. 

Azathioprine and anticalcineurins can be employed as consolidation therapy [41]. In the pediatric cases, mycophenolate mofetil was administered in 9 patients, azathioprine in 7, tacrolimus in 1 patient, and none of the children received cyclosporin A. The selection of therapy may be influenced by individual patient characteristics and their response to initial treatments. 

The prognosis of pediatric anti-GBM antibody disease is contingent upon the promptness of diagnosis and effective management. Oligoanuria remains a critical kidney and vital prognostic factor [42]. In comparison to adults, long-term outcomes in children may be more favorable, with observed renal improvement even in cases of severe kidney impairment (> 90% crescents) necessitating renal replacement therapy during the acute phase. This discrepancy might be attributed to the increased plasticity and potential for regeneration of kidney tissue in children [9, 43]. 

Among the 47 pediatric cases: 34% were able to regain normal renal function. 12,7% evolved into chronic kidney disease. 36% progressed to end-stage renal disease, with 6 of these patients undergoing kidney transplantation. 

Our patient falls into the category of those who regained normal kidney function, representing the 16^th^ such case. Although the recurrence of disease with antibody production has been reported, it is infrequent [9]. Rigorous long-term follow-up is imperative due to the risk of relapse in double-positive patients, necessitating prolonged immunosuppressive treatment. Monitoring CD19 B-cell levels can guide rituximab maintenance therapy, as suggested by the KDIGO recommendations [31]. Regular follow-up and careful monitoring are crucial to identify and manage potential relapses effectively. 

## Conclusion 

Despite the limited literature on pediatric anti-GBM and double-positive disease, it is imperative to consider these diagnoses in pediatric patients presenting with rapidly progressive glomerulonephritis. This case report represents the first documented instance of anti-GBM disease in children in Morocco. Enhanced awareness and understanding of this rare condition are crucial for facilitating prompt diagnosis and implementing appropriate treatment strategies. Preserving renal function emerges as a primary challenge in managing these cases. 

The incorporation of rituximab as an alternative treatment holds promise, offering a potential advantage over other immunosuppressive agents that may pose significant side effects. Continued research and reporting of cases contribute to expanding our knowledge base and refining therapeutic approaches, ultimately improving outcomes for pediatric patients with anti-GBM antibody disease. 

## Funding 

This research received no external funding and was supported by personal funds. 

## Conflict of interest 

The authors have no competing interests. 

## Authors’ contributions 

Nadia Ech-charii and Nabila Chekhlabi conceptualized and designed the study. Rajaa Tissir, Halima Hadri, and Abire Allaoui conducted the literature search. Nezha Dini supervised the study. Nadia Ech-charii and Soukaina Essadiqi wrote the manuscript. All authors have reviewed and approved the final manuscript. 

**Table 1.** Features of the reported pediatric case of anti-glomerular basement membrane disease.

**Author**	**Year**	**Age (years/sex)**	**Initial symptom**	**Pulmonary involvement**	**GFR (mL/min) or creatinine (mg/dL)**	**Kidney biopsy**	**Anti-GBM**	**ANCA ** **type**	**Dialysis**	**IS**	**Plasmapheresis**	**Final outcome**
Our patient	2023	9/M	Fever, pallor, lumbar pain,	–	GFR: 34	Crescents 9/14	+	p-ANCA	HD	MP, CYC, RTX	PE (11)	N, 2 yr
Anjum et al. [51]	2023	10/M	Fever, edema, HU	+	Crea: 4,5	Crescents/ necrosis	+	–	HD	MP, CYC	PE	N.A.
Khan et al. [52]	2022	7/F	HU, PU (SUSP)	_	N	No crescent IF: linear deposits of IgG	+	–	–	MP, CYC	DFPP	N
McAllisterr et al. [53]	2022	14/M	Diarrhea, vomiting, epistaxis, HU	+	Crea: 46,8	Not performed	+	–	HD	MP, CYC, RTX	PE (33)	ESRD RT
Helander et al. [44]	2021	2/F	Sore throat, vomiting, edema, HBP	–	Crea: 5,27	Crescents/ necrosis	+	p-ANCA	HD	MP, CYC, RTX, AZA	PE (15)	ESRD RT 18 months
Sobotta et al. [54]	2021	17/M	Dyspnea, cough, hemoptysis, HU	+	N	Not performed	+	–	–	MP, CYC, Eculizumab	PE (9)	N 6 months
Jen et al. [39]	2021	4/M	Fever, vomiting cough, HU, PU	+	N	No crescent IF: Linear deposits of IgG	+	–	–	MP, RTX, MMF	–	N 9 months
Timmermans et al. [37]	2019	15/F	Dyspnea, cough, thoracic pain	+	Crea: 1,09	Crescents	+	p-ANCA	–	MP, CYC, RTX	PE (14)	N 16 months
Mannemuddhu er al. [55]	2019	15/F	Edema, oliguria, PU, HBP	–	Crea: 5,49	Crescents	+	–	HD	MP, CYC, RTX, AZA	PE (10)	ESRD Died
Şişmanlar-Eyüboğlu et al. [56]	2018	14/M	Fever, malaise, cough, hemoptysis, joint pain	+	N	Not performed	+	–	–	MP, CYC	PE (11)	N 1 yr
Agarwal et al. [45]	2017	11/M	Vomiting, HU, HBP	–	Crea: 7,9	Crescents	+	p-ANCA	HD	MP, CYC	PE (21)	CKD
Raj et al. [57]	2017	2/F	Fever, sore throat, malaise, edema, HU, PU, HBP	+	Crea: 7,01	Sclerosis: 9/12	+	–	HD, PD	MP, CYC, MMF	PE (5)	ESRD, RT
Dorval et al. [36]	2017	7/F	Fever, abdominal pain, cough, headache, HBP, HU	+	GFR: 6,3	Crescents (80%)	+	–	–	MP, CYC	IA (10)	CKD 21 months
Xie et al. [46]	2015	6/F	Edema, oliguria	–	Crea: 9,45	Crescents 100%/ sclerosis	+	p-ANCA	HD	MP, CYC	PE (8)	ESRD
Nagano et al. [24]	2015	8/F	HU, PU (SUSP)	–	N	No crescents IF: linear deposits of IgG	+	–	–	MP, CYC	DFPP (3)	N
O’Hagan et al. [58]	2015	7/M	Abdominal pain, HU, PU, LU	–	GFR: 102	Crescents: 95%	+	–	HD (4)	MP, CYC	PE (19)	CKD 1 yr
Gray et al. [59]	2015	9/M	HU, fever	+	Acute renal failure	Crescents > 90%	+	–		MP, CYC, RTX	PE	CKD
Bogdanovic et al. [21]	2013	10/F	Fever, malaise, leg pain	+	Normal	Crescents/ necrosis	+	p-ANCA	–	MP, CYC, MMF	–	N 10 months
Jiao et al. [60]	2012	15/F	Fever, cough, hemoptysis, oliguria, edema	+	Crea: 11	Sclerosis: 11/12	+	–	HD	MP, CYC	DFPP (3)	ESRD
Bayat et al. [9]	2012	14/F	Pallor, cough, dyspnea, malaise, HU, PU	+	DFG: 60	Crescents	+	–	–	MP, CYC	PE (4)	N
Dalabih et al. [61]	2012	9/F	Respiratory distress, edema, HU, HBP, HPM	+	Crea: 7,35	Crescents	–	–	HD, PD	MP, CYC	PE (6)	ESRD PD
Bjerre et al. [62]	2012	1,5/M	Fever, vomiting, edema, HBP, HU	–	Crea: 1,69	Crescents/necrosis (13/17)	+	–	–	MP, CYC, MMF	PE (13)	N
Williamson et al. [7]	2011	10/M	Pallor, nausea vomiting, cough, HU	–	Crea: 11,5	Crescents (100%)	+	–	PD	MP, CYC, MMF tacrolimus	PE (12)	ESRD RT
Williamson et al. [7]	2010	17/M	Fatigue, edema, HU	+	Crea: 13,9	Crescents (87%)	+	–	PD, HD	MP, CYC, AZA	PE (11)	ESRD HD 1 yr
Williamson et al. [7]	2010	10/M	Fever, cough, hemoptysis, abdominal pain, vomiting, headache, edema	+	Crea: 6,2	Crescents (100%)	+	–	PD	MP, CYC, MMF	PE (6)	ESRD PD 1 yr
Williamson et al. [7]	2010	8/F	Cough, sore throat	+	Crea: 7,7	Crescents (83%)	+	p-ANCA	–	–	–	Died
Dixit et al. [63]	2010	3/F	Fever, pallor, fatigue, edema HU, PU	–	GFR: 23	Crescents: 6/8	+	–	–	MP, CYC, MMF	PE	CKD 3 yr
Poddar et al. [47]	2010	9/M	Fever, vomiting	+	Crea: 8,8	Not performed	+	p-ANCA	HD	MP, CYC	PE (21)	Died 3 months
Naidoo et al. [48]	2009	4/F	Epistaxis, lethargy	–	GFR: 15	Crescents/ sclerosis	+	p-ANCA	–	MP, CYC	PE (11)	CKD 9 months
Hecht et al. [64]	2007	9/F	Abdominal pain, vomiting, malaise, HU, PU	–	Crea: 1,4	crescents (60%)	+	–	–	MP, CYC	PE (13)	N 1yr
Upshaw et al. [65]	2007	16/F	Pallor, lethargy, cough, HBP, HU, PU, LU	+	Crea: 4	Crescents	–	–	–	–	–	NA
Bakkaloglu et al. [66]	2006	5/F	Fever, malaise, joint pain, oliguria, HU, PU	–	Crea: 3,2	Crescents	+	–	PD	MP, CYC, MMF	PE	N 15 months
Hijosa et al. [49]	2005	12/M	Fever, rash, arthritis	+	GFR: 16	Crescents	+	p-ANCA	–	MP, CYC, MMF	PE (10)	CKD 18 months
Gittins et al. [67]	2004	14/F	Pallor, headache, abdominal pain, vomiting, HBP, PU, HU	+	Crea: 19,25	Crescents/necrotic	+	–	HD	MP, CYC	PE	ESRD: HD
Hibbs et al. [68]	2001	4/M	ARF, rash, abdominal pain, vomiting, fever, HU	–	Crea: 8,3	Crescents (100%)	+	–	PD	MP, CYC, AZA	–	ESRD: TR
Filho et al. [69]	2001	10/F	Pallor, hemoptysis	+	GFR: 9,3	NP	+	p-ANCA	PD	–	–	Died
Paueksakon et al. [50]	1999	12/F	Hemoptysis, cough	+	Normal	crescents/necrosis	+	p-ANCA	–	MP, CYC	PE (7)	N
Brito et al. [70]	1997	5/F	Anorexia, lethargy, HU	–	GFR: 39	Crescents	+	–		MP, CYC	PE (15)	N 1 yr
Bigler et al. [71]	1997	0,9/F	–	+	GFR: 5	IF: Linear deposits of IgG	+	–	DP	MP, CYC	PE	ESRD TR
Boven et al. [72]	1996	2/F	Fever, anorexia	–	Crea:4 ,5	Crescents (90%) /necrosis	+	–	PD	MP, CYC	PE (18)	ESRD
McCarthy et al. [73]	1994	10/M	Fever, cough, abdominal pain, vomiting, headache, malaise	+	Crea: 6.2	Crescents (100%) necrosis	+	–	HD	MP, CYC	PE (6)	ESRD TR
Gilvarry et al. [74]	1992	6/M	Abdominal pain, anorexia, lethargy	–	Crea: 3,8	Crescents (70%)	+	–	PD	MP, CYC	PE (14)	N
Harrity et al. [25]	1991	13/F	Cough, fever, weakness, respiratory distress	+	N	Not performed	+	–	–	MP, CYC	PE	Died
Levin et al. [75]	1983	10/F	Vomiting, sore throat, fever, oliguria	–	Creat: 4,5	Crescents (80%)	+	–	DP	MP, AZA, dipyridamole	PE	ESRD HD
Martini et al. [76]	1981	8/F	Pallor, fatigue	+	GFR: 43	Sclerosis I	+	–	HD	MP, CYC	PE	ESRD
Levin et al. [75]	1979	7/F	Diarrhea, vomiting, lethargic, anorexic, pale, anuric	+	Crea: 14,7	Crescents (100%)	+	–	DP	MP, CYC, AZA	PE (16)	ESRD:PD
Levin et al. [75]	1977	4/F	Abdominal pain, pallor, anorexia, HU, PU	–	Crea: 5,27	Crescents (100%)	+	–	–	MP, AZA, CYC	PE	Died

ANCA = anti-neutrophil cytoplasmic antibodies; AZA = azathioprine; ARF = acute renal failure; MP = methyl-prednisolone; Crea = creatinine; CKD = chronic kidney disease; CYC = cyclophosphamide; DFPP = double- filtration plasmapheresis; ESRD = end-stage renal disease; F = female; GN = glomerulonephritis; GFR = glomerular filtration rate; HBP = high blood pressure; HD = hemodialysis; HU = hematuria; HPM = hepatomegaly; IA = immunoadsorption; IS = immunosuppressant; LU = leucocytosis; M = male; MMF = mycophenolate mofetil; N = normal; N.A = not available; NP = not performed; PD = peritoneal dialysis; PE = plasma exchange; PU = proteinuria; RT = renal transplant; RTX = rituximab; SUSP = school urine screening program; Yr = year(s); (+) = positive/present; (–) = negative/absent.
